# Supplementary material for: Transfer of knowledge from model organisms to evolutionarily distant non-model organisms: The coral Pocillopora damicornis membrane signaling receptome
Source: PLoS One. 2023 Feb 3;18(2):e0270965. doi: 10.1371/journal.pone.0270965 (PMC9897584; doi:10.1371/journal.pone.0270965)

**Supplementary Figures and Tables:**

**Figure S1.** Clustal omega multiple sequence alignment of opsin homologs. Residues involved in active site formation are highlighted in blue.


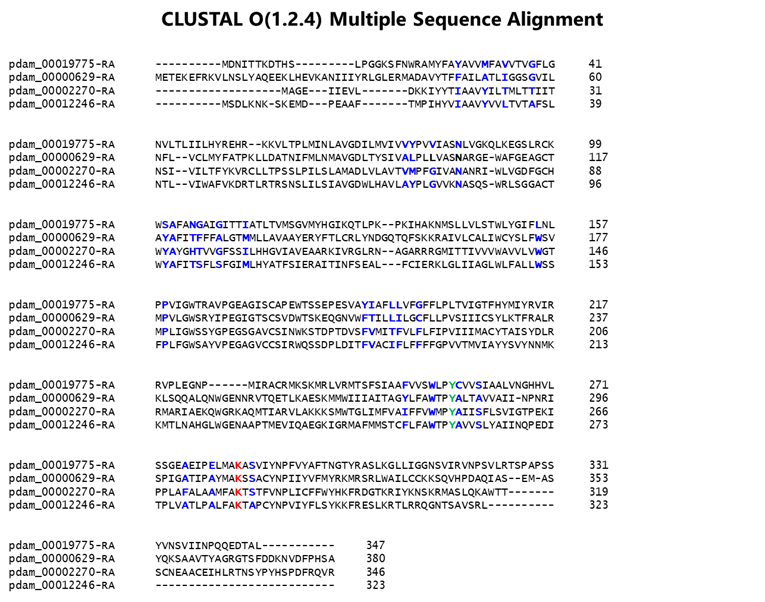

Supplement: S1 Fig — Residues involved in active site formation are highlighted in blue. (DOCX) [file pone.0270965.s001.docx]
