# Supplementary material for: Transfer of knowledge from model organisms to evolutionarily distant non-model organisms: The coral Pocillopora damicornis membrane signaling receptome
Source: PLoS One. 2023 Feb 3;18(2):e0270965. doi: 10.1371/journal.pone.0270965 (PMC9897584; doi:10.1371/journal.pone.0270965)
Supplement: S1 Table — (DOCX) [file pone.0270965.s002.docx]

**Supplementary Table S1.** The details of residues and their molecular interactions with the retinal active site in squid rhodopsin and coral putative rhodopsin proteins.

| **Protein** | **Ligand** | **Covalent bond** | **Pi-Sigma** | **Alkyl and Pi-Alkyl** | **Van der Waals** | **Score**  **kcal/mol** |
| --- | --- | --- | --- | --- | --- | --- |
| Squid Rhodopsin (2ziy) | Retinal | Lys 305 | Phe205, Trp274 | Tyr 111, Val 301, Met204, Phe188, Phe120, Ala278, Phe209 | _ |  |
| Squid Rhodopsin  (2ziy) | Retinal | -- | Phe205, Trp274 | Lys305, Tyr111, Met204, Phe188, Phe120, Ala278, Phe209 | _ | -10.9 |
| 629  Model | Retinal | -- | -- | Ala84, Trp223, Cys169, Phe170, Ala227, Ile166, Trp152, Tyr226, Phe83, Leu85 | _ | -7.6 |
| Model  2270 | Retinal | -- | Trp248 | Phe241, Val191, Phe245, Ala252, Ile244, Ile102, Leu187, Phe99, His94 | _ | -8.4 |
| Model  12246 | Retinal | -- | Phe190 | Phe251, Phe194, Ile189, Ala259, Tyr258, Ala285, Lys286, Ala282, Ile173, Trp255, Phe107 | _ | -8.8 |
| Model  19775 | Retinal | -- | Trp253 | Phe249, Ile110, Ala283, Lys284, Met73, Tyr256, Pro177, Trp179, Cys257, Leu194, Phe198, Leu193 | Gly197, Ile113, Asn156, Gly109, Glu280, Gly106, Asn105, Ala176 | -7.0 |
